# TELEMEDICINE TRENDS IN THREE NURSING HOMES DURING THE COVID-19 PANDEMIC

**DOI:** 10.1093/geroni/igac059.2972

**Published:** 2022-12-20

**Authors:** Lindsay Sandell, Tena Baar, Raza Haque, Angela Zell

**Affiliations:** Michigan State University College of Human Medicine, Grand Rapids, Michigan, United States; Michigan State University, East Lansing, Michigan, United States; Michigan State University, East Lansing, Michigan, United States; Michigan State University, East Lansing, Michigan, United States

## Abstract

Physical distancing and visiting restrictions during the COVID-19 pandemic posed significant challenges in providing timely medical care in nursing homes. The rising number of new COVID-19 cases created hardship in providing scheduled and non-urgent care visits. Virtual visits were pivotal in providing patient care. However, the additional responsibility of facilitating virtual visits for both social and clinical purposes further constrained the ability of nursing home staff to provide care. The critical deficiency of the workforce, rising numbers of new cases, and required triage of patients have been significant barriers to regularly scheduled care. We hypothesize that regular non-COVID-19 services in the evaluated nursing home facilities were lower than COVID-19 visits during this period. Our goal is to show the types of services affected during the pandemic. In this study, we analyzed 563 virtual visits during the COVID-19 pandemic in three nursing home facilities in Michigan from December 2020 through February 2022. Upon analyzing the types of services and trends, our results revealed that the number of COVID-19 related visits (68) was significantly lower than non-COVID-19 related visits (485), refuting our hypothesis. This illustrates that routine care could still be delivered during the pandemic. Additionally, the overall number of virtual visits declined steadily over the study period. This trend could suggest an increase of in-person services or a decrease in COVID-19 cases. The decline could also be related to the barriers faced by the nursing home workforce considering the time and additional responsibility of monitoring a virtual visit.

